# Does mental exertion during incremental exercise change substrate oxidation and cardiorespiratory outcomes in individuals with overweight?

**DOI:** 10.14814/phy2.70172

**Published:** 2025-01-12

**Authors:** Samira Pourmirzaei Kouhbanani, Seyed Kamaledin Setarehdan, Rana Fayazmilani

**Affiliations:** ^1^ Department of Biological Sciences in Sport, Faculty of Sport Sciences and Health Shahid Beheshti University Tehran Iran; ^2^ Control and Intelligent Processing Centre of Excellence, School of Electrical and Computer Engineering, College of Engineering Tehran University Tehran Iran

**Keywords:** cognitive task, graded exercise, substrate oxidation, VO2_max_

## Abstract

Given the growing concern over the impact of brain health in individuals with overweight, understanding how mental exertion (ME) during exercise affects substrate oxidation and cardiorespiratory outcomes is crucial. This study examines how ME impacts these outcomes during an incremental exercise test in adults with overweight. Seventeen adults who were overweight completed an incremental exercise test on a cycle ergometer two times, with and without the Stroop task. Energy expenditure (EE), carbohydrate and fat oxidation, maximum heart rate (HR_max_), maximal oxygen uptake (VO2_max_), maximum fat oxidation (MFO), and the intensity of exercise that elicited MFO (Fat_max_) are measured by indirect calorimetry. ME did not change the EE, carbohydrate, and fat oxidation at any stages of the incremental test. However, ME resulted in significantly lower HR_max_, VO2_max_, and MFO (*p* < 0.01) and increased NASA‐TLX scores but showed no change in Fat_max_. These results show ME decreases the value of HR_max_, VO2_max_, and MFO during the incremental exercise test. Due to the increased mental workload demonstrated by the NASA‐TLX test, adults with overweight are unable to complete the test to the same extent as they did in the test without ME according to maximal levels in this study.

## 1 INTRODUCTION

Overweight and obesity are increasing in different populations and becoming a global public health and economic issue. Studies have shown that some diseases, such as cardiovascular diseases, metabolic syndrome, type 2 diabetes, Alzheimer's disease, and cognitive disorders, are increased due to being overweight and obesity, and it is observed that individuals who are overweight have a higher mortality rate than people of normal weight (Mohajan & Mohajan, 2023). Recently, researchers believe that metabolic imbalances or decreased physical activity may be responsible for significant weight gain (Gómez‐Apo et al., 2021; Oussaada et al., 2019). In contrast, research involving both humans and animals has consistently highlighted the positive impact of physical activity on metabolic processes (De Feo et al., 2003; García‐Giménez et al., 2024; Speakman & Selman, 2003). There is a growing emphasis on investigating how different types of physical activity can influence metabolic rates in the brain. Recent review studies have revealed significant structural changes in the brains of individuals who are overweight (Gómez‐Apo et al., 2021; Herrmann et al., 2019), and some studies have shown that the volume of white and gray matter, as well as noticeable atrophy in the frontal and temporal regions, are negatively correlated with body mass index (BMI), which can impair cognitive function (Cai, 2013; Taki et al., 2008). In addition to BMI, waist circumference and waist‐to‐hip ratio (WHR) are linked to cortical thickness in people who are overweight. The combination of general and central obesity, characterized by WHR greater than 0.85 for women and greater than 0.90 for men, is linked to a reduced volume of gray matter compared to lean adults (Hamer & Batty, 2019; Hayakawa et al., 2018). As a result, individuals with overweight are clinically associated with cognitive disorders, such as disorders of the nervous tissue and a decrease in brain metabolism. Also, thickness reduction may be related to the loss of neurons due to the activation of apoptosis, mitochondrial damage, altered synapses, or neuroinflammation (Gómez‐Apo et al., 2021). Conversely, studies have demonstrated that our brain's demand for oxygen and glucose spikes when engaged in a specific task (motor or cognitive). These events result in an excess blood flow in specific brain areas to meet the increased metabolic needs. Similarly, cerebral blood flow also elevates during physical activity (Park et al., 2021; Perrey, 2008; Steventon et al., 2020). According to these studies, it can be assumed that physical activity alone can increase brain metabolism.

Engaging in mental exertion (ME), such as a cognitive task alone, triggers specific physiological responses associated with hypothalamic–pituitary–adrenal (HPA) and sympathetic‐adrenal (SA) axis activity (Silva‐Júnior et al., 2016). Considering the influence of HPA and SA activity on the cardiopulmonary system, it is conceivable that a combination of mental activity and physical activity may induce cardiopulmonary responses and energy expenditure (EE). Observations have indicated that when engaging in ME induced by a cognitive task alongside sustained physical activity executed at 65% of maximal oxygen uptake (VO2_max_), in subjects with different physical fitness, subjects exhibited elevated heart rate (HR), ventilation, ventilatory equivalent (VE/VO_2_), and RR responses from rest to exercise when faced with a mental challenge in individuals with well‐above‐average fitness compared to those with below‐average fitness (Acevedo et al., 2006). Similarly, in a study where ME is performed along with physical activity, HR, ventilation, breathing rate, and norepinephrine showed a significant increase compared to physical activity alone (Webb et al., 2008). In people with overweight, the impact of dual‐task (cognitive task and exercise) remains unclear; however, older individuals with cognitive impairments have enhanced brain and cognitive function when engaging in such tasks. A meta‐analysis revealed that dual‐task training can improve cognition, physical well‐being, and mood in older adults with cognitive challenges (Ye et al., 2024). Therefore, it is essential to incorporate brain exercises alongside physical training interventions, and further studies in this field are required. Understanding the influence of ME on people with overweight during physical exertion carries significant practical implications.

Fat oxidation predominantly occurs during submaximal intensities (<65% VO2_max_). However, when exercise intensity surpasses 65% VO2_max_, there is a shift in energetic contribution toward carbohydrates (CHO), resulting in the rate of fat oxidation reaching its maximum value (Fat_max_) (Achten et al., 2002). Fat_max_, representing maximal fat oxidation (MFO) during an incremental exercise test, has been suggested as a primary approach for prescribing exercise training when fat oxidation is at its peak (Achten et al., 2002). Furthermore, significant physiological changes were observed in the chess players during a cognitive task such as playing chess for 1 h. These included an increase in HR, a reduction in average HR variability, and a decrease in the respiratory exchange ratio (RER). Also, there is a prominent shift observed toward fat oxidation throughout the physical activity (Troubat et al., 2009). According to these studies, combining mental work and exercise may change oxygen uptake (VO_2_), HR, EE, CHO, and fat oxidation. However, a study has demonstrated that VO2_max_, maximal heart rate (HR_max_), Fat_max_, MFO, CHO, and fat oxidation responses in individuals with normal weight were higher than those with obesity (Dandanell et al., 2017). Previous studies have not explored the effects of a dual‐task on VO2_max_, HR_max_, Fat_max_, and MFO responses in people with overweight. Also, no study has evaluated ME during an incremental exercise to determine substrate oxidation and cardiorespiratory outcomes.

Based on these studies, as well as the intensity changes that occur during the incremental test and lead to changes in substrate oxidation and cardiorespiratory responses (MFO, VO2, Fat_max_, HR_max_, VO2_max_, HR, EE, CHO, and fat oxidation), no studies have been conducted on dual‐task performance in people with overweight. Therefore, this study aims to investigate and compare the two incremental tests with and without ME to measure MFO, Fat_max_, HR_max_, VO2_max_, HR, VO_2_, EE, CHO, and fat oxidation. Currently, cognitive tasks are prevalent in conjunction with physical exercise regimens. Hence, it is imperative to create incremental tests tailored to cognitive functions to gauge these activities' impact, particularly on individuals with overweight, focusing on these variables. Thus, the present study has designed two incremental protocols with dual physical and mental tasks to answer our question.

## 2 MATERIALS AND METHODS

### 2.1 Participants

The sample size was determined using G power (Faul et al., 2007) in conjunction with previously published data (Dandanell et al., 2017), which indicated that a sample size of sixteen was necessary to achieve 95% power and an alpha (*α*) level of 0.05 to detect a significant difference between the two tests. Seventeen healthy, sedentary, and male and female adults with a BMI of >25 kg/m^2^ willingly volunteered to participate in the study (Table 1). The inclusion criteria for participants were as follows: (a) age >20 years; (b) being sedentary (VO2_max_ <35 mL/min/kg) over the last 6 months. All subjects also completed the Beck Physical Activity Questionnaire (BPAQ) to assess their physical activity levels (Baecke et al., 1982). The BPAQ categories include physical activity at work, sports during leisure time, and non‐sport physical activity during leisure time. To be classified as sedentary, participants had to score below the arbitrary cutoff point of 8 from the sum of the three indices (work, sports, and leisure), (c) right hand, (d) non‐smokers, (e) for women, a regular menstrual cycle over the past 6 months was required; (f) no history of psychological disorders, and (g) no acute or chronic disease. The exclusion criteria for participants included: (a) lower limb injury within the previous 2 months, (b) current medication use, and (c) a history of cardiopulmonary, metabolic, or musculoskeletal diseases. Before the tests, participants underwent a pre‐participation screening to gather their medical and training history. The menstrual cycle was not controlled, as previous studies during incremental exercise tests have demonstrated that it does not influence MFO and Fat_max_ (Frandsen et al., 2020). Participant recruitment has been conducted through social media and local media outlets. Participants were thoroughly informed about all experimental procedures, potential risks, and discomforts associated with the experiment before they signed an informed written consent. This study has been approved by the Ethics Committee of Shahid Beheshti University (IR.SBU.REC.1400.268), and the clinical trial has been registered with the code IRCT20221011056149N1 from the Iranian Registry of Clinical Trials. All procedures in this research adhered to the ethical standards set by the institutional research committee and the most recent version of the Helsinki Declaration. Participation was voluntary, and participants had the right to withdraw at any time without penalty.

**TABLE 1:** Descriptive parameters of study participants.

	Mean	SD
Sex, Women/Men	9/8	–
Age (years)	28.2	7.2
Weight (kg)	84.8	14.7
Height (m)	167.2	9.4
Body Mass Index (kg/m2)	29.6	2.6
Body Fat Mass (Kg)	30.7	6.0
Fat mass (%)	38.2	7.4
Waist‐Hip Ratio	0.94	0.05
Heart Rate Rest (beats.min^−1^)	69	9
Resting fat oxidation (g/min)	0.04	0.02

*Note*: All values are expressed in mean ± SD.

### 2.2 Experimental design

This study employed a single‐center, crossover design in which all subjects underwent an incremental exercise test with ME and none ME (NME) on separate occasions. The minimum interval between each occasion was 72 h, while the maximum was 7 days. Participants performed ME during an incremental exercise test in a randomized order in one session. In the other session, subjects performed the incremental exercise test with NME. The MFO and VO2_max_ were measured in two distinct tests, separated by a five‐minute rest period. The MFO, EE, VO_2_, and carbon dioxide production (VCO_2_) were continuously measured using indirect calorimetry to assess substrate oxidation of CHO and fat during rest and throughout the incremental submaximal and maximal exercise tests. The experiment occurred in an exercise physiology laboratory with controlled environmental conditions in a quiet room supervised by the same skilled team.

### 2.3 Pre experimental test (standardizations)

Participants arrived at the laboratory after fasting for 3 h at least (Amaro‐Gahete, Sanchez‐Delgado, Jurado‐Fasoli, et al., 2019; Talanian et al., 2007), and they tracked their dietary intake and beverage consumption for 24 h before they arrived at the laboratory. Additionally, participants were advised to refrain from engaging in moderate to intensive physical activity for 48 h before testing. Participants were encouraged to maintain their physical activity levels and nutritional habits during the tests. They were also requested to avoid consuming caffeine, alcohol, and other stimulants for 12 h before each experimental session and to maintain a consistent sleeping pattern with at least 8 h of sleep before each trial. Self‐reported dietary and exercise records were checked for compliance with these instructions. Before the tests, the body weight, height, fat mass, and BMI were measured using an Inbody 770 scale (model 770, South Korea). Participants also completed the BRUMS questionnaire.

### 2.4 Brunel mood scale

Participants completed the Brunel mood scale (BRUMS) questionnaire (Terry et al., 2003). The questionnaire consists of 24 questions related to emotions and assesses six factors: Anger, confusion, depression, fatigue, tension, and vigor. For each item, participants choose a number from a 5‐point scale where 0 = “not at all,” 1 = “a little,” 2 = “moderately,” 3 = “Quite A Bit,” and “4 = Extremely.” After that, the mood items for each subscale are summed, and a score from 0 to 16 is obtained.

### 2.5 Incremental exercise test

Participants performed an incremental exercise test using an electronically braked cycle ergometer (Monark 834E, Vansbro, Sweden) and a breath‐by‐breath gas analyzer (Metalyzer 3B, Cortex, Germany). The cycle ergometer's saddle and handlebar positions were consistently maintained in all tests. Participants were instructed to remain silent during the test, speaking only in case of emergencies.

**FIGURE 1:**
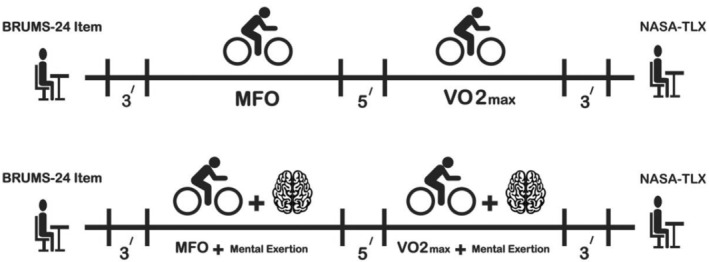
Study procedures. MFO, Maximum fat oxidation; VO2_max_, Maximum oxygen uptake; Mental exertion, Stroop task; (‘), Minutes.

The protocol commenced with a three‐minute resting period. Subsequently, for the MFO test, participants underwent a standardized three‐minute warm‐up on a cycle ergometer at 50 W, maintaining a 60–70 rpm cadence. Following the warm‐up, the intensity increased by 25 W every 3 min until the RER exceeded 1.0. At this point, the intensity was sustained for more than 1 min (Amaro‐Gahete, Sanchez‐Delgado, Jurado‐Fasoli, et al., 2019). Before initiating the maximal incremental exercise test to measure VO2_max_, participants were given a brief five‐minute break with unrestricted access to water. The VO2_max_ test commenced with an initial workload of 50 W for men and 25 W for women, maintaining a cadence of 60–70 rpm for 3 min. Subsequently, workloads increased by 25 W for men and 15 W for women every 2 min. This progression continued until participants either reported reaching their exertion limits or failed to sustain a cadence above 60 rpm. After each stage, participants' fatigue levels were assessed using the 6–20 point Borg scale (Borg, 1990). Another session followed the same protocol but incorporated ME, using the Stroop task during the resting phase, the MFO test, and the VO2_max_ test, as illustrated in Figure 1.

The gas analyzer was calibrated for gas concentration and expired air volume just before the measurements per the manufacturer's guidelines. HR was continuously monitored throughout the test using an HR monitor (N2965, Polar Electro Oy, Kempele, Finland). VO2_max_ was determined based on the final 30 s of the VO_2_ measurement when at least three of the following five criteria were met: (a) a stabilization in VO_2_ (defined as steady <2 mL/min/kg with the increasing workload), (b) RER exceeding than 1.10 (Beltz et al., 2016), (c) rating of perceived exertion above 17 points using the 6–20‐point Borg scale, (d) HR reaching 90% of the age‐predicted maximal HR (i.e., 208–0.7 age) (Heinzmann‐Filho et al., 2018; Yeung et al., 2022), and (e) unable to maintain a cadence above 60 rpm. If these criteria are not fully met, the highest VO_2_ measured over 30 s is recorded as the peak oxygen consumption (VO2_peak_).

### 2.6 Substrate utilization

Values are averaged every 30 s. The average of these variables, taken from the final 30 s of each stage, was utilized for statistical analysis (Amaro‐Gahete, Sanchez‐Delgado, Alcantara, et al., 2019). At each stage, EE, CHO, and fat oxidation rates at rest and during the incremental exercise protocol were calculated using stoichiometric calculations (Frayn, 1983) with the assumption that the urinary nitrogen excretion rate was negligible and protein oxidation was neglected due to the relatively short exercise period. EE, CHO, and fat oxidation rates are calculated as follows:

$$\mathrm{EE}\;\left(\text{kcal}/\min\right)=\left(3.869^{*}{\mathrm{VO}}_{2}\right)+\left(1.195^{*}{\mathrm{VCO}}_{2}\right)$$

$$\mathrm{Fat}\;\text{oxidation}\;\left(g/\min\right)=\left(1.67^{*}{\mathrm{VO}}_{2}\right)–\left(1.67^{*}{\mathrm{VCO}}_{2}\right)$$

$$\mathrm{CHO}\;\text{oxidation}\;\left(g/\min\right)=\left(4.55^{*}{\mathrm{VCO}}_{2}\right)–\left(3.21^{*}{\mathrm{VO}}_{2}\right)$$

where VO_2_ and VCO_2_ are expressed in liters per minute.

The MFO rate is established for each participant as the maximum value of the fat oxidation rate obtained during the incremental exercise test at any stage (Gutiérrez‐Hellín et al., 2021). The present study established MFO and Fat_max_ by utilizing measured values. The obtained MFO has been identified as the peak value of fat oxidation achieved during the submaximal exercise test. The Fat_max_ data were collected during the stage with the highest rate of fat oxidation and also from the corresponding intensity at which VO2_max_ was selected (Amaro‐Gahete, Sanchez‐Delgado, Alcantara, et al., 2019).

### 2.7 Mental exertion

The ME involved a modified computer‐based Stroop Color‐Word Task (SCWT) (Stroop, 1992). Participants received the following instructions: “You will see color words on the screen: yellow, green, blue, and red. Press the color you observe, not the written word. Only read the word when it is red. Each color word is displayed for one second; respond quickly and accurately. We will track the colors you correctly identify.” The test comprised a pseudo‐random sequence, half congruent (word matches color) and half incongruent (mismatched word‐color combinations). In a 34‐point font, each word appeared on a laptop screen for 1 s, followed by a one‐second blank interval before the next word.

### 2.8 NASA task load measures

Upon completion of the exercise, and after a 5‐minute interval, participants were asked to complete the National Aeronautics and Space Administration Task Load Index (NASA‐TLX) rating scale to validate the task's demands and subjective workload. The NASA‐TLX is a multi‐dimensional rating method that provides an overall workload score and assesses six subscales: mental demands, physical demands, temporal demands, performance, effort, and frustration (Hart, 1988). This questionnaire included a 0 to 100‐point scale to assess each item. Participants were previously instructed that a score of 0 meant a minimal amount of that item, while a score of 100 showed its maximal amount.

### 2.9 Statistical analysis

Raw gas exchange parameters (i.e., VO_2_, VCO_2_, EE, CHO, fat oxidation rates, HR, and RER) were downloaded to an Excel spreadsheet and averaged every 30 s. The present study utilized the Shapiro–Wilk test to confirm the normality of all variables and parametric tests to identify differences between tests. Paired *t*‐tests compared variables within the ME and NME for VO2_max_, HR_max_, MFO, and Fat_max_ in incremental exercise protocols. In addition, the Bland–Altman method evaluated the reliability of the outcomes as mentioned above across the two tests. A two (condition) by five (stage) repeated‐measures analysis of variance (RM ANOVA) was performed to analyze the main impact of ME on VO_2_, HR, EE, CHO, fat oxidation, and NASA‐TLX under investigation. After completing the F test to evaluate the main impact of ME at each stage measurement. All statistical tests set a two‐tailed significance level at *p* < 0.05. Descriptive data have been provided as mean ± standard deviation. Data analysis used Statistical Package for Social Sciences (SPSS, v. 26.0, IBM SPSS Statistics, IBM Corporation), and figures were designed using Graph Pad Prism 10.3.1 (Graph Pad Software, LLC, USA).

## 3 RESULTS

Characteristics of the individuals with overweight are reported in Table 1.

**FIGURE 2:**
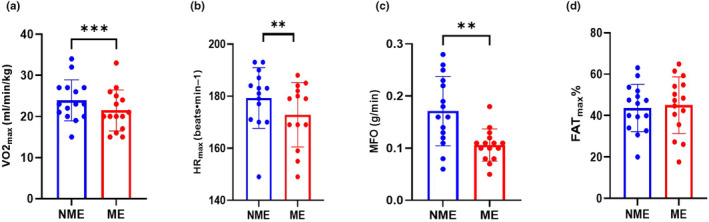
T‐test paired for 15 participants. Maximum oxygen uptake (VO2_max_) (a), Maximum heart rate (HR_max_) (b), Maximum fat oxidation (MFO) (c), and exercise intensity that elicits MFO (Fat_max_) (d) have plotted against exercise intensity (% Maximum oxygen uptake (VO2max)) for the two incremental exercise tests (NME (None Mental Exertion) and ME (Mental Exertion). *p* value obtained by *t*‐test. (**p < 0.01; ***p < 0.001). The data of one female and one male in the ME test and one male were lost in the NME test due to the failure of the HR belt. Also, one missing data point was in VO2_max_, MFO, and fat_max_ for ME and NME tests.

VO2_max_ was notably higher in the NME than in the ME session (23.93 ± 4.99 vs. 21.46 ± 4.98 mL/min/kg, *p* ≤ 0.001, *t* = 5.069, Figure 2a) also just three subjects were in ME, and two subjects were in NME as VO2_peak_. HR_max_ was notably higher in the NME than in the ME session (178 ± 12 beats.min^−1^ vs. 172 ± 12 beats.min^−1^, *p* = 0.001, *t* = 4.390, Figure 2b). Furthermore, a significant difference was observed in the MFO comparison (0.17 ± 0.06 g/min vs. 0.10 ± 0.03 g/min, *p* = 0.002, *t* = 3.923, Figure 2c), and it was considerably higher in the NME test than in the ME test. However, no significant differences have been observed when comparing Fat_max_ (43.66 ± 11.46% vs. 45.00 ± 13.70%, *p* = 0.587, *t* = −0.556, Figure 2d) values in NME vs. ME. It is important to mention that the data of one female and one male in the ME test and one male were lost in the NME test due to the failure of the HR belt. Also, there is missing data points for ME and NME tests due to caffeine consumption by subject which has been excluded from data.

**FIGURE 3:**
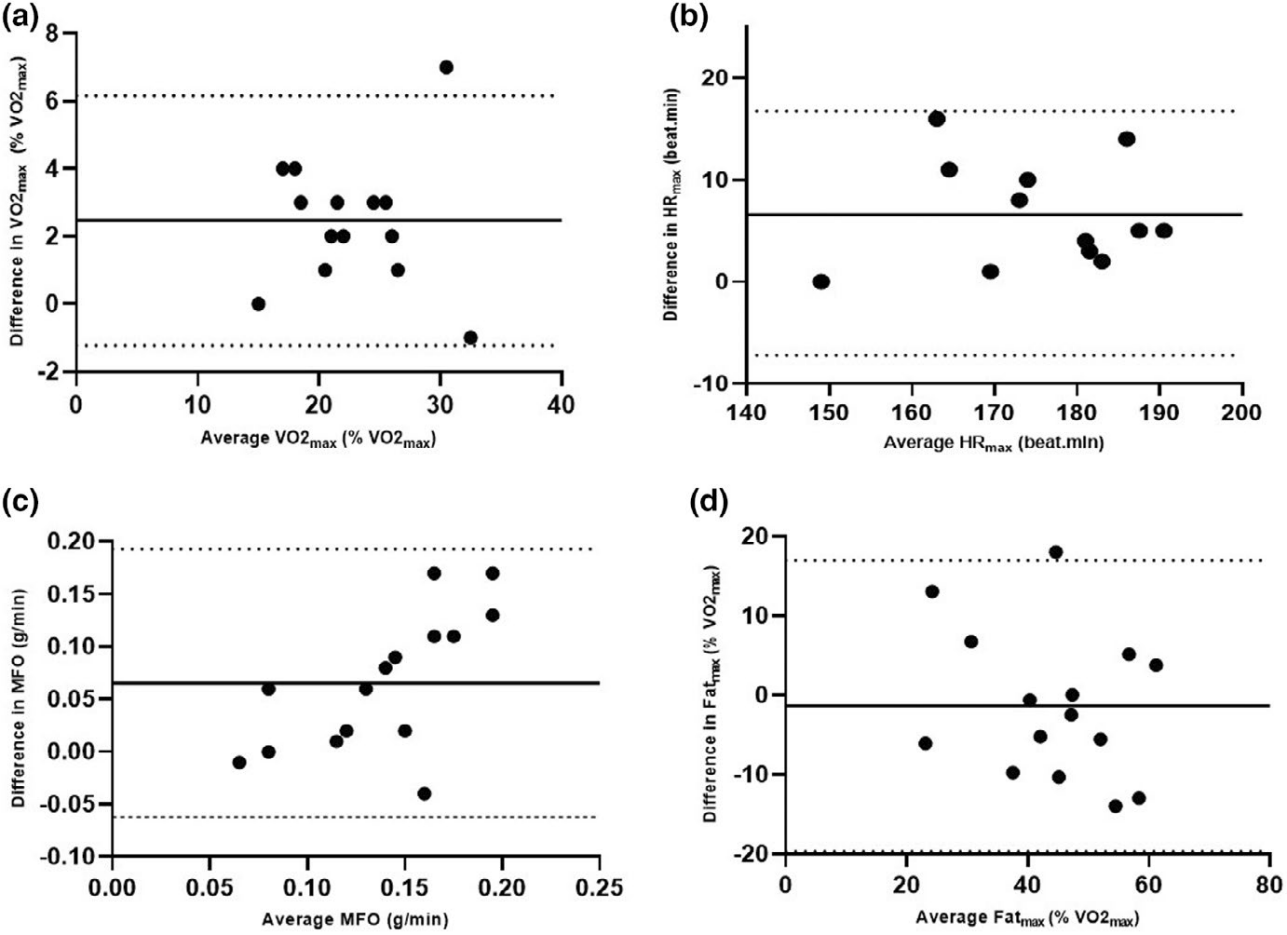
The Bland–Altman plot of the agreement for 15 participants in maximum oxygen uptake (VO2_max_) (a). maximum heart rate (HR_max_) (b), maximum fat oxidation (MFO) (c), and the intensity that elicits MFO (Fat_max_) (d) have been determined with the two incremental exercise tests. Bland–Altman plots with mean differences (solid lines) between direct and 95% limits of agreements (dashed lines) between two tests.

The Bland–Altman plot (Figure 3) presents mean differences and limits of agreement (LoA). In the analysis of agreement between the protocols, there was a bias of 2.46 percentage points of VO2_max_ (95% LoA: −1.22% to 6.16%) for VO2_max_ and 6.58 beats per minute (95% LoA: −3.60 to 16.77 beats per minute) for HR_max_ (Figure 3a–3b). Additionally, the analysis revealed a bias of 0.06 g per minute (95% LoA: −0.06 to 0.19 grams per minute) for MFO and −1.34 percentage points of VO2_max_ (95% LoA: −19.66 to 16.98%) for Fat_max_ (Figure 3c,d).

**FIGURE 4:**
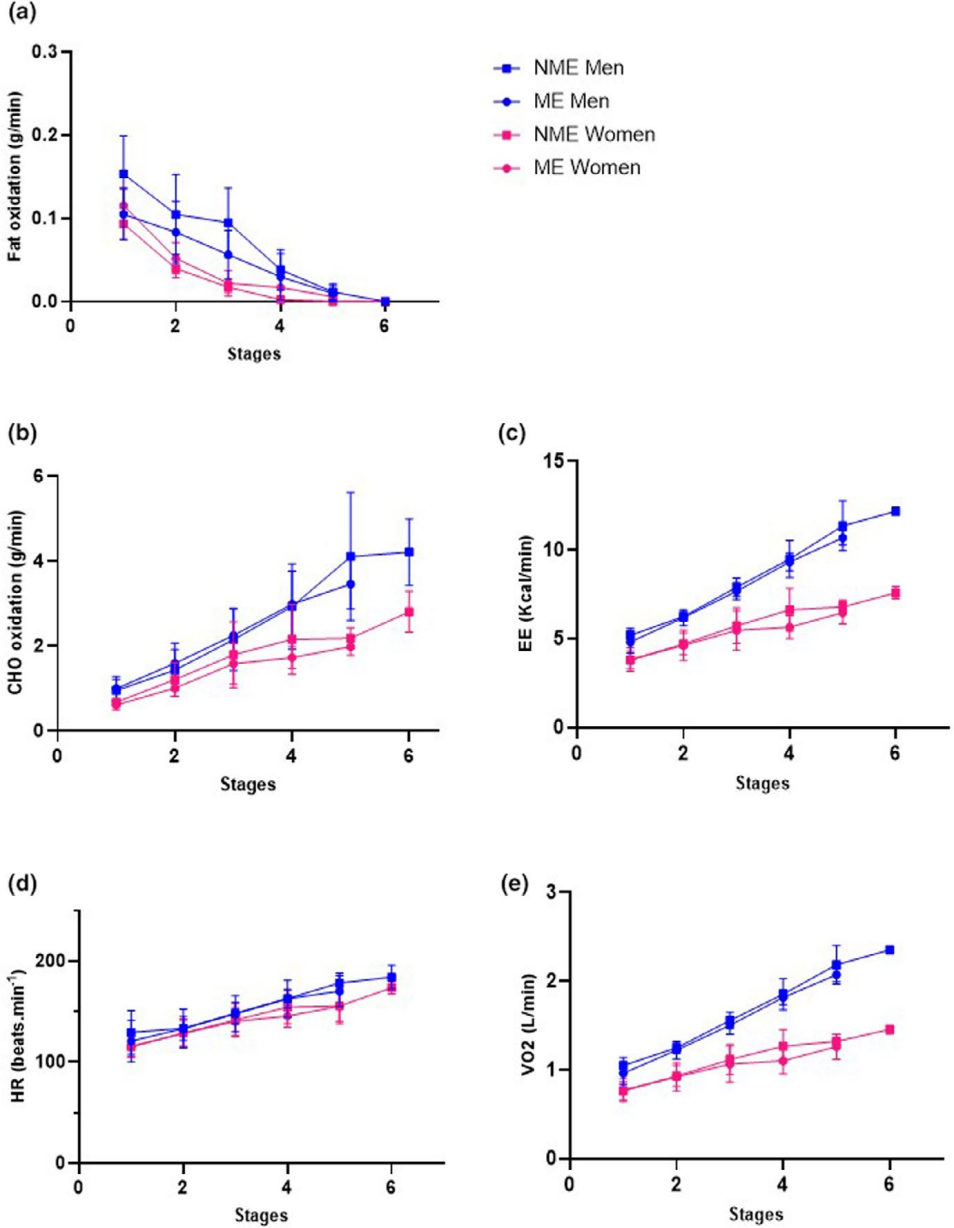
Repeated‐measures analysis of variance for 15 participants. Fat oxidation (a), CHO oxidation (b), energy expenditure (EE) (c), heart rate (d), and oxygen consumption (VO2) (e) have been plotted versus stages for the two incremental tests.

There was no significant effect of ME (session × stage interaction) on fat oxidation rate (*F*_(4,16)_ = 0.529; *p* = 0.716 for women and *F*_(4,16)_ = 1.300; *p* = 0.312 for men, Figure 4a), CHO oxidation rate (*F*_(4,16)_ = 2.297; *p* = 0.104 for women and *F*_(4,16)_ = 1.249; *p* = 0.330 for men, Figure 4b), EE (*F*_(4,16)_ = 3.359; *p* = 0.115 for women and *F*_(4,16)_ = 0.869; *p* = 0.504 for men, Figure 4c), HR (*F*_(4,12)_ = 1.137; *p* = 0.385 for women and *F*_(4,8)_ = 1.423; *p* = 0.310 for men, Figure 4d), and VO_2_ (*F*_(4,16)_ = 2.671; *p* = 0.070 for women and *F*_(4,16)_ = 0.781; *p* = 0.554 for men, Figure 4e) in contrast to NME during exercise in both sex. It should be noted that due to the inability of most subjects to reach the final stages, the two final stages were removed from both tests.

**FIGURE 5:**
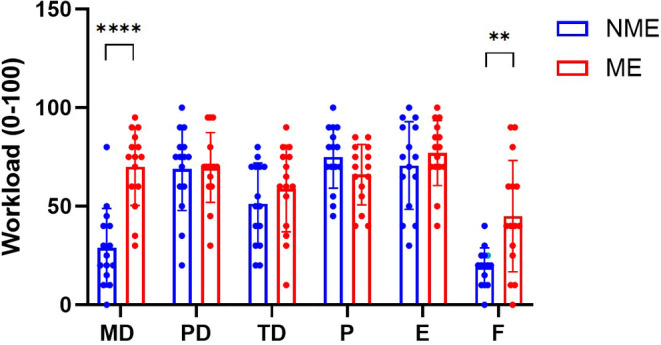
Repeated‐measures analysis of variance for 15 participants using the Bonferroni post hoc test. E, effort; F, frustration; MD, Mental demands; ME, mental exertion; NME, non‐mental exertion; P, performance; PD, physical demands; TD, temporal demands. (**p < 0.01; ****p < 0.0001).

ME had a primary impact on NASA‐TLX scores (*F*_(5,70)_ = 10.78; *p* ≤ 0.001, Figure 5). Perceptions of increased mental demands represented the significant difference in ME (NME 29.00 ± 19.92 and ME 70.00 ± 19.54), as did reports of elevated frustration under the ME scores (NME 19.33 ± 9.61 and ME 45.00 ± 28.22).

## 4 DISCUSSION

The present study aimed to investigate the impact of ME on substrate oxidation and cardiorespiratory outcomes during incremental exercise in adults with overweight. Our results indicate that ME primarily affects maximum physiological values, as seen in the significant reductions in VO2_max_, HR_max_, and MFO when ME was introduced. However, the Fat_max_ remains consistent across both sessions. These findings suggest that while ME does not alter VO_2_, HR, EE, CHO, and fat oxidation at submaximal stages, achieving peak performance is more difficult. The notable decreases in VO2_max_, HR_max_, and MFO under ME likely result from mental fatigue induced by the cognitive load, making it harder for participants to reach their physiological maximum. This is further supported by the increased NASA‐TLX scores, indicating a higher perceived workload in the ME condition. The present study also explores whether this approach could be a reliable method for assessing VO2_max_, HR_max_, MFO, and Fat_max_, specifically for individuals who are overweight, by using Bland–Altman plot analysis. Furthermore, no systematic bias was observed when comparing VO2_max_, HR_max_, MFO, and Fat_max_ between ME and NME conditions. In conclusion, these findings indicate that incorporating ME into incremental exercise can decrease cardiorespiratory outcomes for individuals with overweight.

The rate of CHO and fat oxidation depends on various factors that can alter cellular expression (Purdom et al., 2018). These factors include macronutrient availability in the diet, the type of gas analyzer used, environmental conditions, exercise intensity and duration, fat‐free mass, and fat transport, which can impact CHO and fat oxidation levels during exercise (Amaro‐Gahete, Sanchez‐Delgado, Jurado‐Fasoli, et al., 2019; Purdom et al., 2018; Robles‐González et al., 2021). Recent studies have found evidence of metabolism changes due to mental efforts (Acevedo et al., 2006; Barzegarpoor et al., 2020; Webb et al., 2008). Webb et al. (2008) have noticed changes in the levels of lipolytic hormones such as norepinephrine during physical activity with mental effort. In this study, eight participants performed 37 min of cycling at 60% of VO2_max_ intensity with and without mental work. Our unpublished study has observed that fat oxidation and EE changes during 45‐min moderate exercise increase with the Stroop task and decrease CHO oxidation in people who are overweight. The present study was not significant in VO_2_, HR, EE, CHO, and fat oxidation during exercise, possibly due to increased intensity during incremental tests combined with ME in people who are overweight.

Despite the added mental load, VO_2_, HR, EE, Fat_max_, CHO, and fat oxidation were not affected at submaximal stages, suggesting that while ME impairs overall endurance and peak cardiorespiratory function, it does not disrupt the fundamental metabolic processes involved in substrate utilization at moderate levels of exercise. These findings highlight the critical role of mental fatigue in dual‐task environments, where cognitive and physical demands are combined. The fact that differences were only observed in the maximum values underscores the idea that ME impacts the body's capacity to push to its physiological limits rather than altering performance at submaximal intensity. However, this assumption requires further investigation, particularly in studies focusing on the long‐term effects of moderate intensities, as it remains unclear whether prolonged exposure to ME during moderate exercise could gradually alter substrate oxidation over time. The mental load induced by the cognitive task seems to impose an additional burden, forcing the participants to exert more effort to reach maximal levels of exercise intensity.

Accordingly, previous studies have shown that adding a cognitive load during exercise can impair physical performance, especially at longer durations and time to exhaustion. In one of these investigations, Jost et al. (2023) found that simultaneous cognitive task during exercise increased mental fatigue and reduced physical endurance. This aligns with our findings, suggesting that ME may limit an individual's capacity to reach peak physiological performance by causing early mental exhaustion. In another study, Barzegar pour et al. (2020) investigated the effect of performing prolonged ME during submaximal cycling on mental fatigue, NASA‐TLX, and HR. Exercise tolerance was reduced by adding mental task due to significant subjective fatigue and increased NASA‐TLX scores. However, they did not notice any changes in HR during cycling in both sessions at different times (Barzegarpoor et al., 2020). But other studies have demonstrated changes in HR at various times (Acevedo et al., 2006; Webb et al., 2008). The present study showed increased HR responses for both protocols. However, no significant changes were observed between the tests, suggesting that ME did not significantly impact HR. The authors concluded that extended ME during cycling reduces exercise tolerance, seemingly driven by psychological factors rather than physiological ones. Although there is other study that challenge the idea that ME impairs exercise performance (Holgado et al., 2021). Additionally, it is essential to highlight the characteristics of the subjects in these studies, as none of them were inactive or overweight. Given the brain impairments often observed in individuals with overweight, incorporating cognitive load may further hinder performance in this population (Bischof & Park, 2015). Besides, similar to the differences in maximum values observed in our study, others demonstrate that mental fatigue from prolonged cognitive tasks can decrease maximal physiological outcomes by affecting motivation and perceived effort (Marcora et al., 2009). This is consistent with our results, where ME likely made physical exertion feel more demanding, preventing participants from achieving their maximum cardiorespiratory potential at physical session. The duration and intensity of the physical task appear to be important factors in the decrease in physical performance due to mental effort. The most important factor responsible for the negative impact on physical performance is a higher perceived exertion. Zering et al. (2017) indicated that the VO2_peak_ achieved during the exercise test was significantly reduced after the cognitive task compared to the physical task alone. However, in this study no differences were observed between the conditions in cardiorespiratory variables such as ventilatory threshold and HR_max_. Likewise, HR remained consistent across all iso‐time intervals during the exercise tests. Notably, the rating of perceived exertion was higher at the 50% and 75% iso‐time intervals in the cognitive exertion condition compared to the control condition. In our study, NASA scores were increased, and HR_max_ was lower in the ME test. Also, the MFO is found to be lower in this study in people with overweight than in a previous study with men and women with overweight (Bogdanis et al., 2008). Interestingly, these findings deviate from the results obtained previously. The disparity could be attributed to the utilization of different fasting and physical fitness. This comparison supports the observation that numerous factors influence MFO.

To the best of our knowledge, no studies in the literature have explored the effects of ME on metabolism in individuals with overweight. Significant decreases in MFO, HR_max_, and VO2_max_ have been observed in this research between the conditions. One plausible explanation for the present study's findings is that ME may have reduced physical performance and caused them to become fatigued more quickly by making the work harder so that the participant achieved a lower workload at the end of the incremental test. This could lead to a redistribution of blood flow, consequently decreasing oxygen availability. Such a scenario may limit cognitive performance and brain metabolism (Tempest et al., 2014). The reticular activation hypofrontality theory (RAH) model posits three fundamental energetic principles: (1) the brain receives a stable and finite allocation of resources to meet the energetic demands necessary for neural processing; (2) regions responsible for motor, sensory, and autonomic functions compete for metabolic resources; (3) the balance between cognitive and motor demands influences the distribution of available metabolic resources (Dietrich & Audiffren, 2011). In simultaneous cognitive and physical demands, the competitive nature of neural processing is increased; as a result, the limited available resources lead to decreased activity in some brain areas to reduce the increased demand (for example, physical exercise). Increased cerebral blood flow occurs in “active areas” that are stimulated during cognitive and physical tasks (Audiffren, 2016). As a result, the increase in mental load compared to physical work has reduced physical performance. Dual tasks increase neural demand with increased cerebral oxygenation, but cerebral oxygenation is significantly reduced at near‐maximal exercise intensities. One condition that should be considered in evaluating the studies is understanding how to regulate the blood supply and oxygenation to the brain (Stone et al., 2020) by combining mental tasks and incremental tests.

## 5 LIMITATIONS

Despite the strengths and novelty of the current study, it is essential to interpret the findings with caution. It is crucial to consider the following limitations: (i) The study only included people with overweight, so caution should be exercised in generalizing the findings to other populations, such as elite athletes, normal‐weight adults, patients, or the elderly (ii) Plasma levels of catecholamine, glycerol, and free fatty acid concentrations not assessed. Thus, this study cannot be able to separate the effect induced by ME on adipose tissue/intramuscular triacylglycerol oxidation; (iii) the current study only used one ME at graded exercise for MFO and VO2_max_; it may be that a more stressful stimulus is necessary to obtain higher MFO and HR_max_.

However, these effects had minimal impact on the study outcomes. Nonetheless, the absence of a significant effect of ME on fat oxidation in subjects warrants confirmation in exercise protocols involving continuous exercise, such as 1 h at Fat_max_. However, future research should aim to ascertain whether the effect of acute ME in enhancing or decreasing, VO_2_, HR, EE, CHO, and fat oxidation during exercise of submaximal intensity is consistent across different populations. Further studies are needed to recruit individuals with diverse biological characteristics compared to those included in the present work and employ alternative ME strategies to understand better the differences in HR_max_, VO2_max_, MFO, and Fat_max_.

## 6 CONCLUSION

The results of this study demonstrate that ME during an incremental exercise test reduced MFO, HR_max_, and VO2_max_ in a homogeneous group of individuals with overweight. However, the intensity at which Fat_max_ occurred remained unchanged between the two protocols. ME did not significantly affect substrate oxidation, HR, or EE at submaximal intensities. These findings suggest that ME, when combined with an incremental test, increases mental load as evidenced by changes in NASA‐TLX scores, highlighting the role of mental fatigue in dual‐task settings where both cognitive and physical demands are present. Notably, differences were only observed at maximum values, indicating that ME impacts the body's ability to reach its physiological limits without affecting performance at submaximal intensities. However, this hypothesis requires further investigation, particularly regarding the long‐term effects of moderate‐intensity exercise under ME, as it is still unclear whether prolonged exposure to cognitive exertion during moderate exercise could eventually alter substrate oxidation and overall performance in populations with overweight. The added mental load seems to force participants to expend more effort to reach the maximal level of exercise intensity. This approach can be employed as a load increment for individuals with overweight instead of increasing the physical load as an exercise prescription strategy. Also, the combination of ME and incremental exercise offers a valuable method for assessing MFO and VO2_max_ in dual‐task scenarios. Nevertheless, more research is needed to determine whether these findings apply when using running protocols to estimate MFO and VO2_max_ under ME conditions. Understanding these interactions will be crucial for designing effective exercise interventions that address both physical and cognitive fitness in individuals with overweight.

## FUNDING INFORMATION

This work is based upon research funded by Iran National Science Foundation (INSF) under project No.4012470.

## CONFLICT OF INTEREST STATEMENT

The authors declare that they have no conflict of interest.

## Data Availability

The data that support the findings of this study are available from the corresponding author upon reasonable request.
